# Does the Use of the “Proseek^®^ Multiplex Oncology I Panel” on Peritoneal Fluid Allow a Better Insight in the Pathophysiology of Endometriosis, and in Particular Deep-Infiltrating Endometriosis?

**DOI:** 10.3390/jcm9062009

**Published:** 2020-06-26

**Authors:** Alexandra Perricos, René Wenzl, Heinrich Husslein, Thomas Eiwegger, Manuela Gstoettner, Andreas Weinhaeusel, Gabriel Beikircher, Lorenz Kuessel

**Affiliations:** 1 Department of Obstetrics and Gynecology, Medical University of Vienna, 1090 Vienna, Austria; alexandra.perricos@meduniwien.ac.at (A.P.); heinrich.husslein@meduniwien.ac.at (H.H.); manuela.gstoettner@meduniwien.ac.at (M.G.); lorenz.kuessel@meduniwien.ac.at (L.K.); 2 Department of Pediatrics and Department of Immunology, University of Toronto, Toronto, ON M5G 1X8, Canada; thomas.eiwegger@sickkids.ca; 3 Molecular Diagnostics, Center for Health & Bioresources, AIT Austrian Institute of Technology Vienna, 1190 Vienna, Austria; Andreas.Weinhaeusel@ait.ac.at (A.W.); Gabriel.Beikircher@gmail.com (G.B.)

**Keywords:** endometriosis, multiplex oncology panel, peritoneal fluid

## Abstract

Endometriosis appears to share certain cancer-related processes, such as cell attachment, invasion, proliferation and neovascularization, some of which can also be found in other healthy tissues. In order to better understand the altered milieu of the peritoneal cavity, while acknowledging the reported similarities between endometriosis and neoplastic processes, we applied a multiplex oncology panel to search for specific biomarker signatures in the peritoneal fluid of women with endometriosis, women with deep-infiltrating endometriosis (DIE), as well as controls. In total, 84 patients were included in our study, 53 women with endometriosis and 31 controls. Ninety-two proteins were measured in prospectively collected peritoneal fluid (PF) samples, using the “Proseek^®^ Multiplex Oncology I Panel”. We first compared patients with endometriosis versus controls, and in a second step, DIE versus endometriosis patients without DIE. Out of the 92 analyzed proteins, few showed significant differences between the groups. In patients with endometriosis, ICOS ligand, Endothelial growth factor, E-selectin, Receptor tyrosine-protein kinase erbB-2, Interleukin-6 receptor alpha, Vascular endothelial growth factor receptor 2, Fms-related tyrosine kinase 3 ligand, C-X-C motif chemokine 10, Epididymal secretory protein E4 and Folate receptor-alpha were decreased, while Interleukin-6 and Interleukin-8 were increased compared to controls. Looking at patients with DIE, we found Chemokine ligand 19, Stem cell factor, Vascular endothelial growth factor D, Interleukin-6 receptor alpha and Melanoma inhibitory activity to be increased compared to endometriosis patients without DIE. We have shown a distinct regulation of the immune response, angiogenesis, cell proliferation, cell adhesion and inhibition of apoptosis in PF of patients with endometriosis compared to controls. The specific protein pattern in the PF of DIE patients provides new evidence that DIE represents a unique entity of extrauterine endometriosis with enhanced angiogenetic and pro-proliferative features.

## 1. Introduction

Endometriosis affects 6–10% of women of reproductive age [1] and is characterized by the implantation and proliferation of endometrial tissue outside the uterine cavity [1,2]. Several theories have been established regarding the aetiology of endometriosis, the most widespread of which is Sampson’s theory of retrograde menstruation, which suggests that endometriosis is caused by shed endometrial cells which are washed out through the fallopian tubes into the peritoneal cavity [3]. However, given that retrograde menstruation occurs in the majority of women [4,5,6], it is unclear why only a minority of women develops endometriosis. This underlines the fact that many aspects of the disease still remain unknown.

What we already know about the pathophysiology of endometriosis is that it shares certain cancer-related processes, some of which can also be found in other healthy tissues: Endometrial cells attach to [7,8] and invade [9] tissue of the peritoneal cavity where they induce proliferation [10], neovascularization [11], as well as the invasion of nerve fibers [2]. Moreover, apoptosis plays a key-role in the cyclic changes of eutopic endometrium, allowing monthly decidualization and shedding. The ectopic endometrium in endometriotic lesions underlies similar cyclic changes as eutopic endometrium [7,12]. In order to survive in the peritoneal surrounding, endometriotic cells have developed the ability to escape immune response [13] and a resistance against apoptosis, traits also well known in cancer cells [14]. Although some types of cancer are increased in women with endometriosis, the characteristic pathophysiology of endometriosis leading to an increased incidence of these cancers has low penetration.

There are three subtypes of endometriosis: superficial peritoneal lesions, endometriotic ovarian cysts and deep-infiltrating endometriosis (DIE). DIE, which can be seen as the most severe and aggressive manifestation of endometriosis, is defined by an excessive penetration of the lesions of at least five millimeters into the tissue and most commonly affects uterosacral ligaments, bladder, rectovaginal septum, rectum, and rectosigmoid colon [15].

The clinical presentation of endometriosis can range from asymptomatic patients, to affected women suffering from severe dysmenorrhea, dyspareunia, dysuria, dyschezia and/or subfertility, taking a significant toll on the quality of life [16,17]. The mean interval between the onset of symptoms and obtaining a definitive diagnosis is up to ten years [18]. However, more recent data from the Netherlands demonstrated that median delays of diagnosis for subfertility and pain were 1.75 and 8.33 years, respectively [19]. This calls for better, less invasive screening options, which can only be achieved by a better understanding of the disease in terms of its pathophysiology.

Multiplex technologies offer the ability to test several putative biomarkers simultaneously. Over the past few years, studies have examined the use of this technology in order to investigate the pathophysiology of several diseases with promising results [20,21]. A panel of potential oncological target proteins identified promising biomarkers for the diagnosis of colorectal and ovarian cancer [22,23]. This panel simultaneously analyzed 92 proteins that play a role in a magnitude of cancer-related processes including angiogenesis, apoptotic processes, cell proliferation, immune response, invasion and chemotaxis. Although this method has already been applied in several studies investigating malignant diseases [24,25], very few studies have examined the relevance of multiplex technologies in the field of endometriosis [26,27].

Proseek^®^ Multiplex Oncology I Panel is a reagent kit measuring 92 oncologic disease-related human protein biomarkers. In order to better understand the altered milieu of the peritoneal cavity, while acknowledging the reported similarities between endometriosis and neoplastic processes, we (1) applied the “Proseek^®^ Multiplex Oncology I Panel” to search for specific biomarker signatures in peritoneal fluid (PF) of women with endometriosis compared to controls, and (2) differences between DIE and endometriosis without any sign of DIE.

## 2. Experimental Section

### 2.1. Patients

Samples, collected from 2010 to 2015, were selected from prospectively enrolled patients of the Endometriosis Marker Austria (EMMA) study, a prospective cohort study conducted at the tertiary referral certified Endometriosis Centre of the Medical University of Vienna [28]. The study was approved by the ethics committee of the Medical University of Vienna (EK 545/2010). Written informed consent was obtained from all participants prior to study.

The patients included in our study were premenopausal women between 18 and 50 years of age, who underwent laparoscopic surgery because of suspected endometriosis, infertility, adnexal cysts, chronic pelvic pain of unknown origin, or uterine fibroids. Exclusion criteria included malignant, infectious, or autoimmune diseases.

Baseline characteristics included subjective intensity of pain, notably dysmenorrhea, dyspareunia, dyschezia and dysuria, as well as the influence of pain on the patients’ sex lives (0 = no influence, 10 = maximal influence) using the visual analog scale (VAS), and were obtained preoperatively via a questionnaire. The menstrual cycle phase was histologically determined in eutopic endometrial samples by an experienced pathologist.

In total, 84 patients were enrolled, 53 women with endometriosis, and 31 controls. Patients who were operated on due to benign indications (ovarian cysts, fertility work-up, fibroids) without signs of endometriosis during laparoscopy were defined as controls. In the endometriosis group, all cases were biopsy proven and the disease was classified according to the revised American Fertility Society Score, as mild or minimal (rAFS score I and II) and moderate or severe (rAFS score III and IV). Women with endometriosis were divided into subgroups according to the histologically proven presence (*n* = 19) or absence (*n* = 34) of DIE. In cases of DIE, the ENZIAN score [29] was applied by the surgeon in order to classify the lesions.

### 2.2. Sample Analysis

PF samples were collected prospectively in accordance with the harmonization guidelines [30] after lavage of the peritoneal cavity with 10 mL of sterile 0.9% NaCl. Cell free supernatants were obtained separated by centrifugation (3000 rpm at 4 °C for 10 min) and were stored in aliquots at −80 °C until analysis.

Ninety-two proteins were measured using the “Proseek Multiplex Oncology I v2 96 × 96 Cancer Panel” (OLINK Proteomics, Uppsala, Sweden). A list of all analyzed proteins is shown in Table S1. The measurement was carried out according to the Proseek Multiplex 96 × 96 User Manual.

Specifically, the Proseek reagents are based on Proximity Extension Assay technology, in which 96 oligonucleotide-labeled antibody probe pairs bind to their respective protein targets in the sample. A polymerase chain reaction (PCR) reporter is formed by a proximity-dependent DNA polymerization event which is detected and quantified in real-time PCR [23].

The Fluidigm raw data’s quality was assessed according to OLINK guidelines (Oncology I-v2 Data Preprocessing v1.0, OLINK Proteomics, Uppsala, Sweden). Values in peritoneal fluid were normalized to the total protein amount (measured with the Bradford Assay).

Biostatistical analysis was executed using BRB Array Tools Version 4.4.1 (developed by the National Cancer Institute, National Institute of Health, Bethesda, Rockville, MD, USA) [31] and included class comparison (conducted at a significance level of 0.05), whereby different feature selection criteria were applied. The Normalized Protein eXpression (NPX) values were imported into BRB Tools (National Cancer Institute, National Institute of Health, Bethesda, Rockville, MD, USA), whereby all normalization methods were disabled, since the data were already normalized in the data pre-processing procedure. In our multiplex data analysis, we transformed our linear data in log2 data. Consequently, transforming log2 back to generate linear fold changes, we used the geometric mean for the calculation of the linear “fold change” between the groups [31].

## 3. Results

Patient characteristics are summarized in Table 1.

In two patients, more than 50% of the proteins were under the limit of detection (LOD). Nine proteins were detectable in less than 50% of patients overall, and were therefore excluded from further statistical analysis, as positivity or negativity were not associated with relevant clinical outcomes.

**Figure 1:**
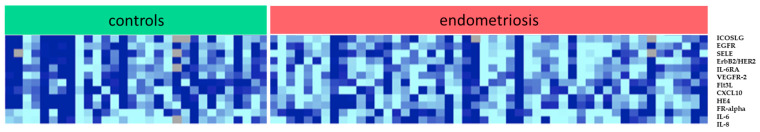
Heat map representing the target proteins that showed a significant difference in expression between patients with endometriosis (DIE and endometriosis patients without DIE) and controls. The color intensity corresponds to the expression level of the protein. DIE, deep-infiltrating endometriosis.

Initially, differential protein expression in endometriosis (*n* = 53) versus controls (*n* = 31) was assessed. Table 2 presents the results of protein expression of the target biomarkers that differed significantly between the two groups. A heat map representation of these proteins is shown in Figure 1.

Twelve biomarkers significantly differed between endometriosis patients and controls. Ten of these biomarkers were significantly lower in endometriosis and two significantly higher in the endometriosis group. Fms-related tyrosine kinase 3 ligand (Flt3L) (*p* = 0.008), Interleukin-6 receptor alpha (IL-6RA) (*p* = 0.022), Epidermal growth factor receptor (EGFR) (*p* = 0.023), Vascular endothelial growth factor receptor 2 (VEGFR-2) (*p* = 0.026), ICOS ligand (ICOSLG) (*p* = 0.027), Epididymal secretory protein E4 (HE4) (*p* = 0.029), C-X-C motif chemokine 10 (CXCL10) (*p* = 0.032), Receptor tyrosine-protein kinase erbB-2 (ErbB2/HER2) (*p* = 0.032), E-selectin (SELE) (*p* = 0.037) and Folate receptor alpha (FR-alpha) (*p* = 0.049) were underexpressed in women with endometriosis. Interleukin-6 (IL-6) and Interleukin-8 (IL-8) however were overexpressed 2.13-fold and 2.56-fold respectively (*p* = 0.045 and *p* = 0.045). These two differentially upregulated biomarkers are related to inflammation, whereas the downregulated markers IL6RA, ICOS ligand and CXCL1 are anti-inflammatory.

**Figure 2:**
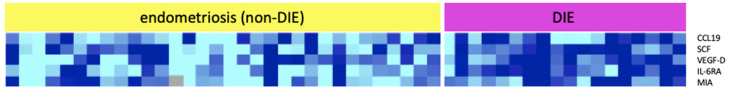
Heat map representing the target proteins that showed a significant difference in expression between endometriosis patients without deep-infiltrating endometriosis and patients with deep-infiltrating endometriosis. The color intensity corresponds to the expression level of the protein.

In a second step, we compared protein expression in women with DIE (*n* = 19) to endometriosis patients without DIE (*n* = 34), of which the statistically different protein expressions are presented in Table 3. A heat map representation of these proteins is shown in Figure 2.

Five biomarkers were significantly overexpressed in patients with DIE compared to endometriosis patients without DIE. In detail, Interleukin 6 receptor alpha (IL-6RA) was overexpressed 1.4-fold (*p* = 0.004), Stem cell factor (SCF) 1.33-fold (*p* = 0.033), Vascular endothelial growth factor D (VEGF-D) 1.75-fold, Chemokine ligand 19 (CCL 19) 1.9-fold (*p* = 0.038), and Melanoma-derived growth regulatory protein (MIA—Melanoma inhibitory activity) 1.32-fold (*p* = 0.040).

## 4. Discussion

Endometriosis exhibits a variety of oncologic features such as invasion [9], proliferation [10], neovascularization [14] and the invasion of nerve fibers [2]. In order to better understand its oncologic-like properties, we applied a multiplex panel with a focus on oncological marker proteins to assess the role of these proteins in the PF of women with endometriosis compared to unaffected controls. We performed two comparative analyses: (1) women with endometriosis vs. controls, (2) women with DIE vs. endometriosis patients without DIE. Although peritoneal and ovarian endometriotic lesions have the capacity to penetrate into the surrounding tissue [32], DIE is characterized by an infiltration of more than five millimeters.

Out of the 92 analyzed proteins, only a few (twelve in the comparison of endometriosis patients and controls and five in the comparison of patients with DIE and endometriosis patients without DIE) showed significant differences between the groups (Table 2 and Table 3). These were proteins involved in immune response (IL-6, IL-8, IL-6Ra, CXCL10, ICOSL, FLT3L, SCF, CCL-19), angiogenesis (IL-8, VEGFR-2, VEGF-D), cell proliferation (EGFR, ErbB2, FR-alpha, ICOSL, HE4, MIA), cell adhesion (SELE), and inhibition of apoptosis (EGFR, ErbB2, ICOSL).

### 4.1. Endometriosis vs. Controls

A summary of characteristics of the significantly increased and depleted proteins in the PF of patients with endometriosis are shown in Table 4.

With regard to the proinflammatory characteristics of endometriosis, our data show some alterations in the PF of women with endometriosis:

Interleukin-6 (IL-6) and Interleukin-8 (IL-8) were the only two proteins found to be significantly increased in patients with endometriosis compared to controls—IL-6 was increased 2.13-fold, and IL-8 was increased 2.56-fold, respectively. In contrast to our data, Rakhila et al. found no significant elevation of IL-6 and IL-8 in their analysis of PF in endometriosis patients compared to controls without endometriosis [46]. It is not clear, however, whether the authors have normalized their data based on the protein content. Several studies support our findings. In concordance with Wang et al., our results show the increased immune response in the peritoneal cavity of endometriosis patients. By activating macrophages, IL-6 can stimulate the proliferation of peritoneal endometriotic lesions [47]. A review by Sikora et al. analyzed the role of IL-8 in the pathophysiology of endometriosis. Several studies have shown a significant increase in IL-8 in the peritoneal fluid of patients suffering from the disease compared to controls, which is also supported by our data. This was suggested to be partly due to a peritoneal increase in other pro-inflammatory factors, such as TNF-alpha and IL-1, in endometriosis patients. It has also been shown that IL-8 is secreted by epithelial cells of eutopic as well as ectopic endometrial tissue [35], and that production of IL-8 is increased in women with endometriosis when stimulated with estrogen and progesterone [48].

The elevated concentrations of IL-6 and IL-8 demonstrate the pro-inflammatory surroundings in the peritoneal cavity of patients with endometriosis.

In addition to these two clearly pro-inflammatory cytokines, ten other proteins showed significantly decreased concentrations in patients with endometriosis compared to controls (Table 2).

The two decreased anti-inflammatory proteins IL-6 receptor alpha (IL-6RA) and ICOSL, in combination with the elevated proinflammatory proteins IL-6 and IL-8, promote and support the highly regulated and dynamic proinflammatory state of the disease [49]. Furthermore, regarding immune response, we found altered levels of additional proteins in PF of endometriosis patients compared to controls. Our results reflect those of a previously conducted study that showed a significant decrease in Flt3L [46].

Both in serum and in PF, CXCL10 has been found to be significantly decreased in advanced stages of endometriosis compared to controls. This fact may contribute to a suppressed T-helper cell response and decreased natural killer cell activity in the PF of affected women. This immune response modulation could facilitate survival of endometriotic cells [50].

Concerning the inhibition of apoptosis and cell proliferation activity of endometriotic cells, we found four factors to be decreased in PF of endometriosis patients compared to controls:

The epidermal growth factor receptor (EGFR) is expressed on many different cell types. The already reported positive correlation between EGFR and Matrix-metalloprotease-7 (MMP-7) seems to play a role in colorectal cancer [51], and EGFR-mediated MMP-7 up-regulation promotes epithelial-mesenchymal transition during ovarian endometriosis progression [52].

The results of an immunohistochemical study analyzing the expression of ErbB2 in endometriotic tissue suggested no significant involvement of this receptor in the pathogenesis of endometriosis [53]. As our results showed a significant decrease in ErbB2 in the PF of endometriosis patients, this supports the theory that ErbB2 does not play a role in the pathogenesis of this disease.

Folate receptor alpha (FR-alpha) seems to play a key role in carcinogenesis, such as in endometrial cancer, whereas in normal and precancerous cells it is of limited expression [54]. Our data therefore seem to support the characteristic of endometriosis as a benign disease.

Human epididymis protein 4 (HE4) may, in combination with CA125, offer new possibilities for noninvasive discrimination between malignant and benign pelvic diseases [43], including endometriosis [55]. The peritoneal fluid of our endometriosis patient collective showed decreased values of HE4.

As all proteins involved in inhibition of apoptosis and cell-proliferation showed either no difference or reduced expression in PF of endometriosis patients, this could suggest a lack of cell-proliferative activity of intraperitoneal endometriotic lesions. The significant decrease in EGFR, ErbB2, FR-alpha, and HE4 in the PF of endometriosis patients compared to controls should be further evaluated.

While angiogenesis is believed to play an important role in the formation of endometriotic lesions, we found the vascular endothelial growth factor receptor 2 (VEGFR-2) to be significantly underexpressed in the PF of affected women compared to controls, and we found no significant difference in its ligand VEGF between the two groups, which does not match the results of previous studies [44].

Proteins involved in cell adhesion processes, such as intracellular adhesion molecule (ICAM) and vascular adhesion molecule (VCAM), seem to be promising biomarkers for endometriosis [8]. However, several studies which analyzed the correlation between E-selectin and endometriosis did not find a significant alteration of its expression in the PF or endometriotic tissue of affected women [56,57]. As we found a significant decrease in E-selectin in endometriosis patients, this cell adhesion molecule does not seem to play a major role in the development of endometriosis.

### 4.2. DIE Patients vs. Endometriosis Patients without DIE

A summary of the characteristics of the significantly increased proteins in the PF of patients with DIE are shown in Table 5.

As our data have demonstrated that proteins involved in immune response are altered in the PF of endometriosis patients compared to controls, we also found further alterations when comparing endometriosis patients with and without DIE:

Stem cell factor (SFC) has been found to be upregulated in the PF of endometriosis patients, as well as in ectopic endometriotic lesions, compared to the eutopic endometrium of endometriosis patients and controls [62,63]. Importantly, the receptor for SCF named c-kit is overexpressed in endometrium of women with endometriosis compared to controls, as well as differently expressed in ectopic lesions depending on their sites, with the highest expression in colorectal lesions (DIE) compared to peritoneal and ovarian endometriosis [58].

An elevated concentration of CCL-19, a chemotactic agent, has also been previously described in PF of women with endometriosis [64]. In line with this, concentration of CCL-19 was found to be elevated in our cohort. CCL-19 was 1.9-fold higher in the DIE, which contributes to endometrial stromal cell invasion and proliferation [65].

In the group of all endometriosis patients we found an elevation of IL-6; however, IL-6RA was significantly decreased compared to controls. In contrast, when we compare DIE to endometriosis patients without DIE, we find the receptor to be overexpressed in DIE patients.

These data suggest that DIE exhibits a more pronounced altered immune response compared the other forms of endometriosis (ovarian and peritoneal).

Regarding the anti-apoptotic and cell-proliferative proteins of this oncologic panel, only Melanoma Inhibitory Activity protein (MIA) showed a significant increase in the PF of DIE patients. In our literature search, we could not find studies dealing with the role of MIA in endometriosis. Therefore, further studies should follow. Our data do not allow the conclusion that deep-infiltrating endometriotic lesions express an enhanced cell-proliferative activity in the peritoneal cavity.

As the involvement of lymph nodes in deep endometriotic lesions has been previously shown, studies have analyzed the role of lymphangiogenetic factors such as VEGF-D and VEGF-C in patients with DIE, and have shown them to be highly expressed in these lesions [61]. Our higher values of VEGF-D in patients with DIE seem to reflect a secretion of VEGF-D of deep-infiltrating endometriotic lesions into PF and, thus, lymphangiogenic properties.

In theory, endometriosis exhibits properties similar to oncologic diseases; therefore, the results of our study may be surprising, given the relative moderate number of biomarkers which were upregulated. This may support the idea that the peritoneal milieu does not seem to fully reflect the oncologic pattern of endometriosis compared to controls, and represents an environment with a much higher level of regulation, yet still with a significant clinical phenotype. A limitation of our study is certainly the restriction of soluble factors that are surrogate markers of cellular activation and interact with receptors on cells which are not represented in this dataset. Despite that, we were able to identity five proteins in the PF of patients with DIE which were enriched in comparison to endometriosis patients without DIE. These findings support the theory that DIE, the most severe form, displays distinct aspects of micro-regulation which are characterized by anti-inflammatory pro-proliferative features. A strength of our study is the prospective cohort design of well-characterized endometriosis patients and controls with the retrieval of peritoneal fluid by standardized washing. In combination with a multiplex analysis of 92 proteins, our data offer a more detailed insight into whether the mechanisms which are known from oncologic diseases are also altered in endometriosis.

## 5. Conclusions

While the pathophysiology of endometriosis has not yet been fully elucidated, applying the Proseek^®^ Multiplex Oncology I Panel clearly demonstrates distinct regulations of the immune response, angiogenesis, cell proliferation, cell adhesion and inhibition of apoptosis in the PF of patients with endometriosis compared to controls. In addition, we found a DIE-specific protein pattern compared to endometriosis patients without DIE. This provides new evidence that DIE represents a unique entity of extrauterine endometriosis with enhanced angiogenetic and pro-proliferative features. The altered milieu of PF in patients with endometriosis compared to controls, and the inconsistency of already published data in combination with the not-fully understood pathophysiology of this enigmatic disease, open new fields for further investigation.

## Figures and Tables

**Table 1:** Patient characteristics.

Patient Characteristics	Control Group (*n* = 31)	Endometriosis (*n* = 53)	*p*-Value	Endometriosis without DIE (*n* = 34)	Endometriosis with DIE (*n* = 19)	*p*-Value
**General Information**						
Age (years)	34.3 ± 6.0	33.1 ± 6.2	0.277	33.5 ± 6.0	32.4 ± 6.6	0.486
BMI (kg/m^2^)	26.2 ± 6.5	22.5 ± 4.0	0.006	23.2 ± 4.5	21.4 ± 2.7	0.540
Gravidity	1.5 ± 1.7	0.5 ± 1.1	<0.001	0.7 ± 1.3	0.2 ± 0.5	0.161
Parity	0.5 ± 0.7	0.3 ± 0.8	0.039	0.4 ± 0.9	0.2 ± 0.5	0.173
**Preoperative pain symptoms**						
Dysmenorrhea (*n*, %)	30 (96.8%)	50 (94.3%)	0.613	33 (97.1%)	17 (89.5%)	0.290
Dysmenorrhea Intensity (VAS range 0–10)	6 (4–8)	8 (6–10)	0.003	8 (6–10)	8 (5.5–10)	0.917
Dyspareunia (*n*, %)	16 (51.6%)	27 (50.9%)	0.935	16 (47.1%)	10 (52.6%)	0.697
Dyspareunia Intensity (VAS range 0–10)	6 (4.25–8)	6 (4–8)	0.414	6 (4.25–8)	5 (4–7.25)	0.917
Influence of pain on Sex Life (*n*, %)	12 (38.7%)	20 (37.7%)	0.929	11 (32.4%)	8 (42.1%)	0.478
Influence of pain on Sex Life Intensity (VAS range 0–10)	4.5 (3.23–8.75)	6 (4–10)	0.915	7 (5–10)	5.5 (2.5–8)	0.917
Cycle Phase			0.528			0.983
Proliferative (*n*, %)	13 (41.9%)	26 (49.1%)		16 (47.1%)	9 (47.4%)	
Secretory (*n*, %)	18 (58.1%)	27 (50.9%)		18 (52.9%)	10 (52.6%)	
rAFS score (*n*, %)						
I	NA	10 (18.9%)		9 (26.5%)	1 (5.3%)	
II	NA	9 (17%)		8 (23.5%)	1 (5.3%)	
III	NA	17 (32.1%)		10 (29.4%)	7 (36.8%)	
IV	NA	17 (32.1%)		7 (20.6%)	10 (52.6%)	
Endometrioma (*n*, %)	NA	32 (60.4%)		23 (67.6%)	9 (47.4%)	0.148
ENZIAN score						
A 1–3	NA	12 (22.6%)	NA	NA	12 (63.2%)	NA
B 1–3	NA	18 (34.6%)	NA	NA	18 (94.7%)	NA
C 1–3	NA	7 (13.2%)	NA	NA	7 (36.8%)	NA
FA	NA	0	NA	NA	0	NA
FI	NA	1 (1.9%)	NA	NA	1 (5.3%)	NA
FO	NA	0	NA	NA	0	NA

DIE: deep-infiltrating endometriosis, BMI: body mass index, VAS: visual analogue scale, rAFS: revised American Fertility Society, NA: not applicable, FA: adenomyosis, FI: bowel endometriosis (cranial of the rectosigmoid junction), FO (“other”): endometriosis of other locations.

**Table 2:** Significant results in the comparison of target protein concentration between endometriosis patients and controls. The fold change describes the ratio of protein expression values between the two tested groups.

	Target	Geometric Mean of Intensities in		Fold Changein Endometriosis	*p*-Value
		Controls (*n* = 31)	Endometriosis (*n* = 53)		
Total	ICOSLG	0.72	0.56	0.78	0.027
	EGFR	1.20	0.99	0.83	0.023
	SELE	1.63	1.29	0.79	0.037
	ErbB2/HER2	7.27	5.81	0.80	0.032
	IL-6RA	2.35	1.87	0.80	0.022
	VEGFR-2	3.88	3.14	0.81	0.026
	Flt3L	37.77	28.17	0.75	0.008
	CXCL10	41.80	24.43	0.58	0.032
	HE4	68.25	42.36	0.62	0.029
	FR-alpha	30.51	18.25	0.60	0.049
	IL-6	16.55	35.53	2.13	0.045
	IL-8	5.69	14.52	2.56	0.045
Proliferative Cycle Phase (*n*, %)		13 (41.9%)	26 (49.1%)		
Secretory Cycle Phase (*n*, %)		18 (58.1%)	27 (50.9%)		

ICOSLG, ICOS ligand; EGFR, Epidermal growth factor receptor; SELE, E-selectin; ErbB2/HER2, Receptor tyrosine-protein kinase erbB-2; IL-6RA, Interleukin-6 receptor alpha; VEGFR-2, Vascular endothelial growth factor receptor 2, Flt3L, Fms-related tyrosine kinase 3 ligand; CXCL10, C-X-C motif chemokine 10; HE4, Epididymal secretory protein E4; FR-alpha, Folate receptor alpha; IL, Interleukin.

**Table 3:** Significant results in the comparison of target protein concentration between patients with DIE and endometriosis patients without DIE. The fold change describes the ratio of protein expression values between the two tested groups.

	Target	Geometric Mean of Intensities in		Fold Change in DIE	*p*-Value
		Non-DIE (*n* = 34)	DIE (*n* = 19)		
Total	CCL 19	13.97	26.53	1.90	0.038
	SCF	5.57	7.42	1.33	0.033
	VEGF-D	4.23	7.42	1.75	0.034
	IL-6RA	1.66	2.33	1.40	0.004
	MIA	0.94	1.24	1.32	0.040
Proliferative Cycle Phase (*n*, %)		16 (47.1%)	9 (47.4%)		
Secretory Cycle Phase (*n*, %)		18 (52.9%)	10 (52.6%)		

CCL 19, Chemokine ligand 19; SCF, Stem cell factor; VEGF-D, Vascular endothelial growth factor D; IL-6RA, Interleukin-6 receptor alpha; MIA: Melanoma inhibitory activity; DIE: deep-infiltrating endometriosis.

**Table 4:** Summary of the characteristics of factors that are significantly enriched or depleted in endometriosis patients compared to controls.

Target	Involved in:	Relation of Factor in Endometriosis Compared to Controls	Characteristics
Interleukin-6 (IL-6)	IR	↑	- during inflammation → local synthesis and release into bloodstream - pleiotropic effects on hematopoiesis and immune response. - induces production of acute-phase-proteins and leads to an increased formation of T-helper cells and cytotoxic T cells [33] - overexpressed in many types of cancer cells → regulates anti-apoptosis, cell survival, proliferation, invasion, cancer-cell metabolism [34]
Interleukin-8 (IL-8)	IR, AG	↑	- chemokine, mainly secreted by macrophages and monocytes - chemotactic agent for neutrophils, as well as for a subset of T-lymphocytes - stimulator for angiogenesis [35]
Interleukin-6 receptor alpha (IL-6Ralpha)	IR	↓	- membrane associated subunit of IL-6 receptor → cis signaling pathway → transcription of inflammatory molecules [36]
Inducible Co-Stimulator Ligand (ICOSL)	IR, AA, CP	↓	- inducible T-cell costimulator and as such plays an important role in the inflammatory response - promotes cell survival, proliferation and differentiation [37] - oncogenesis: anti-tumor T cell response/pro-tumoral response through induction of immunosuppressive Treg cell activity
FMS-like tyrosine kinase 3 ligand (FLT3L)	IR	↓	- cytokine involved in dendritic cell development and therefore critical for the immune response [38]
C-X-C motif chemokine C (CXCL10)	IR	↓	- chemokine, attracts inflammatory leukocytes to the site of inflammation - found to play a role in cancer development, attracting cancer cells to sites of metastatic spreads [39]
Epidermal growth factor receptor (EGFR)	AA, CP	↓	- binding of a ligand → autophosphorylation of EGFR → cell proliferation - triggered pathways may lead to apoptosis-resistance, invasion into the surrounding tissue and metastases [40]
ErbB2/HER2	AA, CP	↓	- part of the human epidermal growth factor receptor - important in the pathogenesis of breast cancer → promotes cell proliferation and survival with anti-apoptotic pathways [41]
Folate receptor alpha (FR-alpha)	CP	↓	- high binding-affinity for the active form of folate - overexpressed in certain tumors such as ovarian, breast and lung - may promote cancer-cell growth [42]
human epididymis protein 4 (HE4)	CP	↓	- potential marker for malignant gynecological diseases - overexpressed in ovarian cancer relative to normal tissue [43]
Vascular endothelial growth factor receptor-2 (VEGFR-2)	AG	↓	- crucial mediator for angiogenesis [44]
Endothelial Selectin (SELE)	CA	↓	- selectins: cell adhesion molecules involved in inflammatory and angiogenic reactions - endothelial selectin (E-selectin) expressed on inflamed vessels → vascular adhesions [45]

IR: immune response, AG: angiogenesis, CP: cell proliferation, CA: cell adhesion, AA: anti-apoptosis. ↓: decreased in endometriosis compared to controls, ↑: increased in endometriosis compared to controls.

**Table 5:** Summary of the characteristics of factors that are significantly enriched in patients with DIE compared to endometriosis patients without DIE.

Target	Involved in:	Relation of Factor in DIE Compared to Non-DIE	Characteristics
Stem cell factor (SCF) (c-kit ligand)	IR	↑	- growth factor, promotes development and differentiation of hematopoietic progenitor cells - promotes the proliferation, survival and maturation of mast cells [58]
Interleukin-6 receptor alpha (IL-6R-alpha)	IR	↑	- membrane associated subunit of IL-6 receptor → cis signaling pathway → transcription of inflammatory molecules [36]
Chemokine ligand 19 (CCL-19)	IR	↑	- promotes migration of certain cells of the immune system, in particular antigen-presenting dendritic cells [59]
Melanoma inhibitory activity (MIA)	CP	↑	- melanoma derived growth-regulatory protein - expressed in malignant melanomas - important for the cellular invasion and development of metastases if this disease [60]
Vascular endothelial growth factor D (VEGF-D)	AG	↑	- involved in lympangiogenesis - may play a role in lymphatic metastases [61]

IR: immune response, AG: angiogenesis, CP: cell proliferation. ↑: increased in DIE patients compared to endometriosis patients without DIE.

## Supplementary Materials

The following are available online at https://www.mdpi.com/2077-0383/9/6/2009/s1, Table S1: List of all proteins included in the Proseek^®^ Multiplex Oncology I v2 96 × 96 Cancer Panel.
