# Supplementary material for: Does the Use of the “Proseek® Multiplex Oncology I Panel” on Peritoneal Fluid Allow a Better Insight in the Pathophysiology of Endometriosis, and in Particular Deep-Infiltrating Endometriosis?
Source: J Clin Med. 2020 Jun 26;9(6):2009. doi: 10.3390/jcm9062009 (PMC7355450; doi:10.3390/jcm9062009)
Supplement: Supplementary file 1 [file jcm-09-02009-s001.pdf]

Adrenomedullin (AM)<br><br>Amphiregulin (AR)<br><br>Angiopoietin-1 receptor (TIE2)<br><br>B-cell activating factor (BAFF)<br><br>Cadherin-3 (CDH3)<br><br>Carbonic anhydrase IX (CAIX)<br><br>Carcinoembryonic antigen (CEA)<br><br>Caspase-3 (CASP-3)<br><br>C-C motif chemokine 19 (CCL19)<br><br>CD40 ligand (CD40-L)<br><br>C-X-C motif chemokine 5 (CXCL5)<br><br>C-X-C motif chemokine 9 (CXCL9)<br><br>C-X-C motif chemokine 10 (CXCL10)<br><br>C-X-C motif chemokine 11 (CXCL11)<br><br>C-X-C motif chemokine 13 (CXCL13)<br><br>Cyclin-dependent kinase inhibitor 1 (CDKN1A)<br><br>Cystatin-B (CSTB)<br><br>Early activation antigen CD69 (CD69)<br><br>Epidermal growth factor receptor (EGFR)<br><br>Epididymal secretory protein E4 (HE4)	Ezrin (EZR)<br><br>Fas antigen ligand (FasL)<br><br>FAS-associated death domain protein (FADD)<br><br>Fms-related tyrosine kinase 3 ligand (Flt3L)<br><br>Folate receptor alpha (FR-alpha)<br><br>Follistatin (FS)<br><br>Furin (FUR)<br><br>Growth hormone (GH)<br><br>Growth/differentiation factor 15 (GDF-15)<br><br>Heparin-binding EGF-like growth factor (HB-EGF)<br><br>Hepatocyte growth factor (HGF)<br><br>ICOS ligand (ICOSLG)<br><br>Immunoglobulin-like transcript 3 (ILT-3)<br><br>Integrin alpha-1 (ITGA1)<br><br>Interferon gamma (IFN-gamma)<br><br>Interleukin-1 receptor antagonist protein (IL-1ra)<br><br>Interleukin-2 (IL-2)<br><br>Interleukin-6 (IL-6)<br><br>Interleukin-6 receptor subunit alpha (IL-6RA)<br><br>Interleukin-7 (IL-7)<br><br>Interleukin-8 (IL-8)	Latency-associated peptide transforming growth factor beta-1 (LAP TGF-beta-1)<br><br>Lipopolysaccharide-induced tumor necrosis factor- alpha factor (LITAF)<br><br>Macrophage colony-stimulating factor 1 (CSF-1)<br><br>Matrix metalloproteinase-1 (MMP-1)<br><br>Melanoma-derived growth regulatory protein (MIA)<br><br>MHC class I polypeptide-related sequence A (MIC-A)<br><br>Midkine (MK)<br><br>Monocyte chemotactic protein 1 (MCP-1)<br><br>Myeloid differentiation primary response protein MyD88 (MYD88)<br><br>NF-kappa-B essential modulator (NEMO)<br><br>NT-3 growth factor receptor (NTRK3)<br><br>Ovarian cancer-related tumor marker CA 125 (CA-125)<br><br>Parkinson disease protein 7 (PARK7)<br><br>Placenta growth factor (PlGF)<br><br>Platelet endothelial cell adhesion molecule (PECAM-1)<br><br>Platelet-derived growth factor subunit B (PDGF subunit B)<br><br>Prolactin (PRL)

Epithelial cell adhesion molecule (Ep-CAM)	Interleukin-12 (IL-12)	Prostasin (PRSS8)
Erythropoietin (EPO)	Interleukin-17 receptor B (IL-17RB)	Receptor tyrosine-protein kinase erbB-2 (ErbB2/ HER2)
E-selectin (SELE)	Kallikrein-11 (hK11)	Receptor tyrosine-protein kinase erbB-3 (ErbB3/ HER3)
Eukaryotic translation initiation factor 4B (eIF-4B)	Kallikrein-6 (KLK6)	Receptor tyrosine-protein kinase erbB-4 (ErbB4/ HER4)
Extracellular matrix metalloproteinase inducer (EMMPRIN)	Tumor necrosis factor ligand superfamily member 14 (TNFSF14)	Regenerating islet-derived protein 4 (REG-4)
Stem cell factor (SCF)	Tumor necrosis factor receptor 1 (TNF-R1)	Urokinase plasminogen activator surface receptor (U-PAR)
Tartrate-resistant acid phosphatase type 5 (TR-AP)	Tumor necrosis factor receptor 2 (TNF-R2)	Vascular endothelial growth factor A (VEGF-A)
Thrombopoietin (THPO)	Tumor necrosis factor receptor superfamily member 4 (TNFRSF4)	Vascular endothelial growth factor D (VEGF-D)
Tissue factor (TF)	Tumor necrosis factor receptor superfamily member 6 (FAS)	Vascular endothelial growth factor receptor 2 (VEGFR-2)
TNF-related apoptosis-inducing ligand receptor 2 (TRAIL-R2)	Tyrosine-protein kinase Lyn (LYN)	Vascular endothelial statin (VE-statin)
Transforming growth factor alpha (TGF-alpha)	Tyrosine-protein phosphatase non-receptor type 22 (PTPN22)	Vimentin (VIM)
Tumor necrosis factor (TNF)		

Table S1: List of all proteins included in the Proseek® Multiplex Oncology I v2 96x96 Cancer Panel.
